# Impact of sentinel lymph-node biopsy and FDG-PET in staging and radiation treatment of anal cancer patients

**DOI:** 10.1038/s41598-020-71577-8

**Published:** 2020-09-03

**Authors:** Najla Slim, Paolo Passoni, Elena Incerti, Roberta Tummineri, Calogero Gumina, Giovanni Mauro Cattaneo, Paola De Nardi, Carla Canevari, Claudio Fiorino, Monica Ronzoni, Andrea Marco Tamburini, Valentina Burgio, Luigi Gianolli, Nadia Di Muzio

**Affiliations:** 1 Department of Radiation Oncology, IRCCS San Raffaele Scientific Institute, Milan, Italy; 2 Unit of Nuclear Medicine, IRCCS San Raffaele Scientific Institute, Milan, Italy; 3 University of Milano-Bicocca, Milan, Italy; 4 Unit of Medical Physics, IRCCS San Raffaele Scientific Institute, Milan, Italy; 5 Unit of Gastroenterology Surgery, IRCCS San Raffaele Scientific Institute, Milan, Italy; 6 Department of Medical Oncology, IRCCS San Raffaele Scientific Institute, Milan, Italy

**Keywords:** Positron-emission tomography, Anal cancer

## Abstract

To assess the role of sentinel lymph-node biopsy (SLNB) and FDG-PET in staging and radiation treatment (RT) of anal cancer patients. This retrospective study was performed on 80 patients (male: 32, female: 48) with a median age of 60 years (39–89 years) with anal squamous cell carcinoma who were treated from March 2008 to March 2018 at the IRCCS San Raffaele Hospital. Patients without clinical evidence of inguinal LNs metastases and/or with discordance between clinical evidence and imaging features were considered for SLNB. FDG-PET was performed in 69/80 patients. Patients with negative imaging in inguinal region and negative SLNB could avoid RT on groin to spare inguinal toxicity. CTV included GTV (primary tumour and positive LNs) and pelvic ± inguinal LNs. PTV1 and PTV2 corresponded to GTV and CTV, respectively, adding 0.5 cm. RT dose was 50.4 Gy/28 fractions to PTV2 and 64.8 Gy/36 fractions to PTV1, delivered with 3DCRT (n = 24) or IMRT (n = 56), concomitant to Mitomycin-C and 5-FU chemotherapy. FDG-PET showed inguinal uptake in 21/69 patients (30%) and was negative in 48/69 patients (70%). Lymphoscintigraphy was performed in 11/21 positive patients (4 patients SLNB confirmed inguinal metastases, 6 patients false positive and 1 patient SLN not found), and in 29/48 negative patients (5/29 showed metastases, 23/29 true negative and 1 SLN not found). Sensitivity, specificity, positive and negative predictive value of FDG-PET were 62%, 79%, 40% and 82%, respectively. Median follow-up time from diagnosis was 40.3 months (range: 4.6–136.4 months): 69 patients (86%) showed a complete response, 10 patients (13%) a partial response, 1 patient (1%) a stable disease. Patients treated on groin (n = 54) versus not treated (n = 26) showed more inguinal dermatitis (G1–G2: 50% vs. 12%; G3–G4: 17% vs. 0%, p < 0.05). For patients treated on groin, G3–G4 inguinal dermatitis, stomatitis and neutropenia were significantly reduced with IMRT against 3DCRT techniques (13% vs. 36%, p = 0.10; 3% vs. 36%, p = 0.003; 8% vs. 29%, p = 0.02, respectively). SLNB improves the FDG-PET inguinal LNs staging in guiding the decision to treat inguinal nodes. IMRT technique significantly reduced G3-G4 toxicities when patients are treated on groin.

## Introduction

Squamous cell carcinoma (SCC) of the anal canal is a relatively uncommon tumour constituting only 4% of all lower alimentary tract and 2% of all digestive system malignancies. The tumour size (T stage) and the nodal status are recognized as the main prognostic factors^1^. Chemo-radiotherapy (CRT) is the standard treatment for anal SCC; whereas mesorectal and internal iliac lymph nodes (LNs) are routinely included in the radiation fields, the need of prophylactic inguinal irradiation is controversial.

Accurate staging is crucial for radiotherapy (RT) treatment planning, aiming to select patients with localized disease. Fifteen-25% of patients with anal cancer show inguinal LNs metastases, while 44% of all LNs with positive metastases were reported to be smaller than 5 mm^2^ and this is the main reason explaining the low sensitivity of either clinical examination in the detection of positive inguinal LNs.

In a retrospective analysis of 270 patients who did not receive prophylactic inguinal irradiation, after a median follow-up of 72 months, the incidence of inguinal metastases was 8%^3^. Two retrospective studies reported the inguinal recurrence rate in patients staged with conventional examinations who did not receive prophylactic inguinal irradiation: Blinde et al.^4^ showed inguinal recurrence rate of 0% in T1, 10% in T2 and 20% in T3–T4 patients with a median follow-up of 65 months, and Ortholan et al.^5^ reported an inguinal recurrence rate of 12% for T1–T2, and 30% for T3–T4 patients with a median follow-up of 61 months. As a consequence, the prescription of prophylactic inguinal irradiation based only on the T stage would cause an unnecessary irradiation of about 70–80% of T3–T4 patients, submitting them to a risk of severe acute toxicity, especially when using conformal RT techniques^6^. Conversely, about 10% of T1-T2 patients would be undertreated if inguinal irradiation is not prescribed. Thus, a better detection of initial inguinal LNs involvement would be important for selecting those patients who really deserve the inguinal irradiation. Recently, FDG-PET was shown to detect more abnormal inguinal LNs than clinical^7^, ultrasound inguinal^8^ or CT^9–11^ examination while sentinel lymph-node biopsy (SLNB) has been shown to be superior to both CT and FDG-PET in detecting metastatic LNs^12^.

Prophylactic RT including inguinal LNs irradiation was shown to decrease inguinal metastasis in several series^3,5^ but is unavoidably associated with increased toxicity. Better patient selection and the use of advanced RT techniques, such as intensity modulated RT (IMRT) and image guided RT (IGRT), may reduce toxicity^13^.

In the present study we reported our experience about the role of SLNB compared to FDG-PET in terms of disease staging and the clinical impact of inguinal irradiation with the use of advanced RT techniques.

## Methods and materials

### Characteristics of patients

Eighty patients with histologically proven anal SCC treated between March 2008 and March 2018 were enrolled in a retrospective study. Patients’ ages ranged from 39 to 89 years with a median of 60 years. Of these 32 (40%) were male and 48 (60%) were female. Only 27/80 (34%) were HIV positive, and 21/27 (78%) were male. The main characteristics of patients are summarized in Table 1.

**Table 1:** Patient characteristics (n = 80 patients).

N. patients (%)	80 (100%)
**Gender**	
Male	32 (40%)
Female	48 (60%)
Median age (range)	60 (39–89)
**Histological subtype**	
Squamous	80 (100%)
**Localization**	
Anal canal	56 (70%)
Anal canal + rectal	12 (15%)
Anal canal + margin	12 (15%)
T1	14 (18%)
T2	30 (37%)
T3	24 (30%)
T4*	12 (15%)*
	*8: vagina
	1: uterus
	3: prostate
N0	29 (36%)
N1	17 (21%)
N2	14 (18%)
N3	20 (25%)
M0	73 (91%)
M1*	7 (9%)*
	*3: external iliac
	2: liver (1: liver + bone)
	2: retroperitoneal
**HIV status**	
Positive	27 (34%) F: 6 (22%); M: 21 (78%)
Negative	53 (66%) F: 42 (79%); M: 11 (21%)

Patients underwent complete workup with full physical examination (anorectal, vaginal, and inguinal sites), proctoscopy or colonoscopy, chest-abdomen CT and pelvic MRI. Patients with T1N+, T2–T4 with any N, M0, Karnofsky performance status ≥ 70, adequate bone marrow, renal and hepatic function were candidate to CRT. TNM staging was T1 in 14, T2 in 30, T3 in 24 and T4 in 12 patients due to involvement of vagina (n = 8), prostate (n = 3) and uterus (n = 1). N staging was N0: 29, N1: 17, N2: 14, and N3:20 patients.

Seven patients with limited metastatic disease were enrolled after collegial discussion and approval, despite the protocol violation (3 patients: external iliac LNs, 2 patients: liver (1 patient: liver + bone), 2 patients: retroperitoneal LNs). The protocol was approved by our Institutional Ethical Committee (IRCCS San Raffaele Hospital Clinical Research Office), and all patients signed an informed consent. A retrospective comparison between FDG-PET inguinal LNs evaluation and SLNB results was made.

### SLNB protocol and treatment

Patients without clinical evidence of inguinal LNs involvement or with discordance between diagnostic imaging and clinical examination were considered for the SLNB protocol. The SLNB is a minimally invasive procedure which consists in submucosal injection of Technetium-99 labelled radio colloid around the primary tumour, patients in whom inguinal tracer uptake is detected undergo radio guided surgical removal of the inguinal sentinel LNs.

The methods relative to lymphoscintigraphy, surgical removal and histopathological examination of sentinel LNs have been already described^14,15^. Patients after positioning in supine position on Comby-Fix^®^ underwent a contrast-enhanced CT and FDG-PET simulation. FDG-PET was used both for staging and target definition. CTV included the GTV (primary tumour and any positive LNs), the ischiorectal fossa, the mesorectum, and the internal and common iliac LNs until the L5-S1 space.

As the inguinal LNs irradiation is the standard treatment in anal cancer, all patients enrolled in this study with positive SLNB were treated on groin. Patients who had negative SLNB and negative imaging in inguinal region were considered for RT without groin irradiation, so as to spare inguinal toxicity; patients were also involved in the final clinical decision. PTV1 and PTV2 were defined as GTV and CTV, expanded with a margin of 0.5 cm, respectively. Median prescribed dose was 50.4 Gy in 28 fractions (1.8 Gy/fraction) to the PTV2, and 64.8 Gy in 36 fractions, delivered as sequential or concomitant boost, to the PTV1. RT techniques consisted of 3DCRT or IMRT (Volumetric Modulated Arc Therapy (VMAT) Rapid Arc or Helical Tomotherapy). The planned concomitant chemotherapy schedule was continuous infusion 5-FU 1,000 mg/mq^2^ delivered from day 1 to 4 and from day 29 to 32 combined to Mitomycin-C 10 mg/m^2^ delivered on day 1 to 29.

### Analyses

Sensitivity, specificity, area under the curve (AUC), positive and negative predictive values of FDG-PET against SLNB were assessed in the subgroup of patients that performed both examinations.

Acute toxicities were scored according to the NCI-CTC for Adverse Events (Version 3); treatment interruptions were also reported. In order to assess the gain in avoiding to treat the inguinal LNs, a comparison in terms of inguinal LNs outcome and toxicity was made between the inguinal RT “IRT” and non-inguinal RT “NIRT” groups, also taking into account RT technique (3DCRT vs. IMRT).

The differences between proportions (“IRT” vs. “NIRT”, 3DCRT vs. IMRT) were tested by the two-tailed Fisher exact test.

Time to progression (TTP) was calculated from the end of treatment and overall survival (OS) from the time of diagnosis to progression, last follow-up or death. Survival outcomes were evaluated using the Kaplan–Meier method. Statistical analyses were performed using MedCalc software (Version 12.1.4, MedCalc, Ostend, Belgium).

### Ethical approval

All procedures performed in studies involving human participants were in accordance with the ethical standards of the Institutional Research Committee and with the principles of the 1964 Declaration of Helsinki and its later amendments or comparable ethical standards.

### Informed consent

Informed consent was obtained from all individual participants included in the study.

## Results

### Comparison between FDG-PET data and SLNB

Sixty-nine/80 patients (86%) underwent FDG-PET that showed inguinal uptake in 21/69 patients (30%). Lymphoscintigraphy was performed in 11/21 (52%) patients: SLNB confirmed inguinal metastases in 4/10 patients (40%), 6/10 patients (60%) were false positive and SLN was not found in 1 patient. FDG-PET was negative in 48/69 patients (70%): lymphoscintigraphy was performed in 29/48 PET-negative patients: 5/28 (18%) showed metastasis despite the PET result, 23/28 (82%) were true negative and SLN was not found in 1 patient. The comparison between FDG-PET inguinal uptakes and SLNB is reported in Table 2.

**Table 2:** (Crude data) Diagnostic accuracy of PET against SLNB evaluation (n = 69 patients).

	PET 69/80 (86%)	Positive inguinal PET: 21/69 (30%)	Negative inguinal PET: 48/69 (70%)
With SLNB	40/69 (58%)	11/21 (52%)	29/48 (60%)
Available SLNB information	38/69 (55%) (2 not found)	10/21 (48%) (1 not found)	28/48 (58%) (1 not found)
Positive SLNB	9/38 (24%)	4/10 (40%) TP	5/28 (18%) FN
Negative SLNB	29/38 (76%)	6/10 (60%) FP	23/28 (82%) TN

PET: positron emission tomography; SLBN: sentinel lymph-node biopsy; TP: true positive; TN: true negative; FP: false positive; FN: false negative.

The resulting sensitivity, specificity, positive and negative predictive value of FDG-PET were 62%, 79%, 40% and 82%, respectively as reported in Table 3.

**Table 3:** Diagnostic statistics (in bracket the 95% confidence intervals are shown).

AUC	Sensitivity	Specificity	Positive predictive value	Negative predictive value
0.68 (0.46–0.85)	62% (35%–5%)	79% (55%–93%)	40% (19%–85%)	82% (69%–98%)

### Treatment feasibility

All patients received the planned dose of 50.4 Gy in 28 fractions to the pelvis and ischiorectal fossa. The median dose of boost to T stage was 64.8 (61.4–72.4 Gy). Fifty-four/80 patients received prophylactic (n = 34) or curative (n = 20) inguinal irradiation (the “IRT” group) and 26/80 patients did not (the “NIRT” group). Boost was delivered sequentially in 96% of patients and concomitant in the remaining 4%. The median dose to the “IRT” group was 50.4 Gy (50.4–70.2 Gy). Twenty-five/26 patients of the “NIRT” group underwent SLNB procedure: 24/25 patients with histologically negative SLNB (2/24 patient with FDG-PET, MRI and CT negative), and 1/25 SLN not found.

In the “NIRT” group the percentage of patients with T1-T2 disease was higher than the percentage of patients with T3–T4 (73% vs. 27%, p = 0.002), while in the “IRT” group the rates were similar (53% vs. 47%, respectively), as reported in Table 4.

**Table 4:** Patient characteristics in “IRT” vs. “NIRT” groups.

	All patients (%)	Inguinal RT “IRT (%)	No inguinal “NIRT” (%)
Patients	80 (100)	54 (68)	26 (32)
Male	32 (40)	22 (41)	10 (38)
Female	48 (60)	32 (59)	16 (62)
**Localization**			
Anal canal	56 (70)	37 (68)	19 (73)
Anal canal + rectal	12 (15)	8 (15)	4 (15)
Anal canal + margin	12 (15)	9 (17)	3 (12)
T1	14 (17)	8 (15)	6 (23)
T2	30 (38)	17 (32)	13 (50)
T3	24 (30)	18 (33)	6 (23)
T4	12 (15)	11 (20)	1 (4)
N0	29 (36)	15 (28)	14 (54)
N1	17 (21)	11 (20)	6 (23)
N2	14 (18)	12 (22)	2 (8)
N3	20 (25)	16 (30)	4 (15)
M0	73 (91)	48 (89)	25 (96)
M1	7 (9)	6 (11)	1 (4)
Positive PET/negative PET	0/49	18/30	2/19
Positive SLNB/negative SLBN	12/34	12/13	0/21
3DCRT	24 (30)	14 (26%)	10 (39)
Tomotherapy	18 (23)	14 (26%)	4 (15)
VMAT	38 (47)	26 (48)	12 (46)

PET: positron emission tomography; SLBN: sentinel lymph-node biopsy; TP: true positive; TN: true negative; FP: false positive; FN: false negative; VMAT: volumetric modulated arc therapy; IRT: inguinal RT; NIRT: non inguinal RT.

For N staging we took into account any information from CT, MRI and FDG-PET. In the “NIRT” group the percentage of N0-N1 was higher than the percentage of N2–N3 (77% vs. 23%, p = 0.0003), while in “IRT” they were similar (52% vs. 48%), as reported in Table 4.

Mean duration of RT was 58 days (36–88 days): 42 patients (53%) needed a temporary suspension of RT (“IRT”: 29 patients and “NIRT”: 13 patients). Mean duration of suspension was 11 days (2–38 days); the mean duration in “IRT” was 11 days (2–38 days) and 12 days (3–23 days) in “NIRT”. Mean dose to suspension: 37.7 Gy (16.2–55.8 Gy) (“IRT”: 34.4 Gy (16.2–55.8 Gy) and “NIRT”: 44.7 Gy (34.2–57.6 Gy)).

RT was delivered with 3DCRT in 24/80 patients (30%), 14/24 patients in the “IRT” group and 10/24 patients in the “NIRT” group. Fifty-six/80 (70%) patients were treated with IMRT: 18/80 patients (22%) were treated with Tomotherapy, (“IRT”: 14/18; “NIRT”: 4/18); 38/80 patients (47%) with VMAT (“IRT”: 26/38; “NIRT”:12/38).

Concomitant Mitomycin-C and 5-FU was prescribed for 71/80 patients (89%), 2/80 patients (2.5%) received only Capecitabine and other 2/80 patients (2.5%) received Cisplatine. Median cycle of chemotherapy was 2 cycles (1 to 4 cycles). The 5/80 remaining patients (6%) who did not received concomitant chemotherapy: 3/5 patients were affected by hepatic cirrhosis and 2/5 patients were > 80 years old.

### Toxicity

There was no significant difference between G1–G2 and G3–G4 toxicities in “IRT” group compared to “NIRT” group except for inguinal dermatitis, as expected (G1–G2: 27/54 patients (50%) vs. 3/26 patients (12%), p = 0.001; and G3–G4: 9/54 patients (17%) vs. 0/26 patients (0%) (p = 0.03)). Toxicity data are reported in Table 5. When comparing 3DCRT vs. IMRT, for diarrhoea and stomatitis the differences in term of G1–G4 toxicities were statistically significant (21/24 (88%) vs. 26/56 (46%), p < 0.01; and 11/24 (46%) vs. 2/56 (4%), p < 0.01). Moreover, in patients treated on groin, IMRT showed reduced G3–G4 inguinal toxicity (5/40 (13%) vs. 5/14 (36%), p = 0.10) stomatitis (1/40 (3%) vs. 5/14 (36%), p = 0.003) and neutropenia (3/40 (8%) vs. 4/14 (29%), p = 0.02) compared to 3DCRT, as reported in Table 6.

**Table 5:** Toxicity evaluation in “IRT” vs “NIRT” groups (n = 80 patients), p < 0.05.

Toxicity	Inguinal RT “IRT” 54/80 (%)		No inguinal RT “NIRT” 26/80 (%)	
	G1–G2	G3–G4	G1–G2	G3–G4
Perineal dermatitis	30 (56)	22 (41)	12 (46)	14 (54)
Inguinal dermatitis	27 (50)* (p = 0.001)*	9 (17)^#^ (p = 0.03)^#^	3 (12)*	0^#^
Diarrhoea	25 (46)	3 (6)	17 (65)	2 (8)
Genito-urinary	19 (35)	1 (2)	13 (50)	0
Vaginitis	6/32 (19)	0	5/16 (31)	0
Nausea/vomiting	13 (24)	0	3 (12)	0
Stomatitis	4 (7)	2 (4)	3 (12)	0
Neutropenia	10 (19)	8 (15)	5 (19)	5 (19)

IRT: inguinal RT; NIRT: non inguinal RT.

* Inguinal G1-G2 dermatitis “IRT” vs “NIRT”.

^#^Inguinal G3–G4 dermatitis “IRT” vs “NIRT”.

**Table 6:** RT techniques toxicity evaluation in “IRT” vs. “NIRT” groups (n = 80 patients), p < 0.05.

Toxicity	3DCRT: 24/80				IMRT: 56/80			
	IRT 14/24 (%)		NIRT 10/24 (%)		IRT 40/56 (%)		NIRT 16/56 (%)	
	G1–G2	G3–G4	G1–G2	G3–G4	G1–G2	G3–G4	G1–G2	G3–G4
Perineal dermatitis	6 (43)	6 (43)	4 (40)	6 (60)	22 (55)	16 (40)	6 (38)	8 (50)
Inguinal dermatitis	(29)	5 (36)^§^ (p = 0.10)^§^	1 (10)	0	22 (55)	5 (13)^§^	1 (6)	0
Diarrhoea	11 (79)	1 (7)	7 (70)	2 (20)	14 (35)	2 (5)	10 (63)	0
Genitourinary	5 (36)	0	4 (40)	0	13 (33)	0	7 (43)	0
Vaginitis	3/9 (33)	0	2/7 (29)	0	3/23 (13)	0	3/9 (33)	0
Nausea/vomiting	4 (29)	0	2 (20)	0	9 (23)	0	1 (6)	0
Stomatitis	3 (21)	5 (36)* (p = 0.003)*	3 (30)	0	1 (3)	1 (3)*	0	0
Neutropenia	2 (14)	4 (29)^#^ (p = 0.02)^#^	5 (50)	1 (10)	9 (23)	3 (8)^#^	0	4 (25)

IRT: inguinal RT; NIRT: non inguinal RT; IMRT: intensity modulated RT.

^§^G3–G4 inguinal dermatitis in IRT: 3DCRT vs IMRT.

* G3–G4 stomatitis in IRT: 3DCRT vs IMRT.

^#^G3–G4 neutropenia in IRT: 3DCRT vs IMRT.

### Outcome

Sixty-nine/80 patients achieved the complete response, 10/80 patients had a partial remission and 1/80 patient had stable disease. Nineteen/80 patients had progression disease, 8/19 had local relapse (5/8: only local relapse, 3/8: local + distant relapse), and 11/19 had only distant relapse. No patient treated or not on groin experienced inguinal relapse. Sixty-four/80 patients are alive and 16/80 patients died for progression disease at the last follow-up.

Median time to local relapse was 38.9 months (4.8–123.2 months). Median time to progression was 35.2 months (3.0–133.0 months), excluded patients with metastasis at the time of diagnosis. Median OS from the time of diagnosis was 40.3 months (4.6–136.4 months), 46/80 patients (58%) achieved a 3 years OS and 32/80 patients (40%) were alive at 5 years from the end of treatment. Nine/80 patients underwent surgery (7/9 abdominal-perineal amputation, 2/9 tumour resection), mean time to surgery was 12.1 months (3.1–23.3 months).

## Discussion

CRT is the standard treatment for anal SCC. The indication of prophylactic inguinal irradiation is, however, controversial. According to the two largest retrospective studies^4,5^ it is clear that treating or not the inguinal LNs based only on the T stage has a large risk of over/under-treatment. A better detection of early inguinal LNs involvement could properly select patients who really deserve inguinal irradiation.

FDG-PET detects more abnormal inguinal LNs than clinical examination^7^, inguinal ultrasound^8^, and CT^9–11^, and, more in general, changing the TMN stage in 41% of patients with anal cancer; thus it has been recommended for the initial staging^16^. The low incidence of metachronous metastases and the side effects after radiotherapy could not justify a prophylactic treatment. A refined staging system with precise identification of disease extent could allow individualized therapy, ensuring the accurate coverage of disease while sparing disease-free organs. Feasibility and efficacy of SLNB has been addressed by several reports and the clinical utility of this procedure in improving disease staging and selecting patients for inguinal radiation and changing the therapeutic plan has also been outlined^14,15^.

To our knowledge, only the study by Mistrangelo et al.^1^ compared FDG-PET and SLNB in 27 patients. Pathologic inguinal uptake was found in 7 patients. SLNB confirmed inguinal metastases in 3/7 (42%) vs. our 4/10 (40%) patients, with 4/7 (57%) false positives vs. 6/10 (60%) in our patients, and did not show inguinal metastases in the 20 patients with no pathologic inguinal uptake. They reported positive and negative predictive value equal to 43% and 100% respectively, quite consistent with the values found in current study (40% and 82%, respectively (Table 3), although, differently from our findings which showed 18% of false PET negative they did not report any false PET negative.

Engledow et al.^17^ reported pathologic inguinal uptake in 9/40 patients. Fine needle aspiration or conventional histology confirmed inguinal metastases in 7/9 (78%) patients. Despite the relatively small number of patients of these studies and of our, results are quite consistent and show that: a) despite recommended for initial staging, the FDG-PET results on inguinal LNs should be interpreted with caution, showing a large number of false positives; b) SLNB can further improve inguinal staging reducing FDG-PET false positive and false negative rates. In the present study FDG-PET importantly showed a false positive rate of 60% (6/10 patients), and a false negative rate of 18% (5/28 patients) when compared to SLNB.

For these reasons, SLNB can be considered as a good tool when comparing new modalities such as FDG-PET.

In particular, the reduction of the false negative rate could be very important, especially in case of stage T1-T2, as a positive SLNB would possibly suggest the radiation oncologist to treat the groins even in presence of a negative FDG-PET. Mistrangelo et al.^12^ reviewed the literature and found 6 studies reporting SLNB positivity rate stratified by T stage: summing his results to the data from all these studies, SLNB found metastases in 6/27 (22%) T1 patients and in 17/98 (17%) T2 patients. In our population, selecting T1 and T2 patients without considering N stage, SLNB found metastasis in 2/14 (14%) and 6/30 (20%) patients, respectively. Considering patients with T1 and T2 N0 stage at diagnostic imaging, who would probably be selected to skip inguinal RT, SLNB showed metastases in 1/9 (11%) and 1/12 (8%) patients, respectively. In short, with the limitation of the small number of patients in the present study and in literature, SLNB seems to be the most effective procedure to select patients for inguinal RT.

Interestingly, with a median OS of 48.5 months (1.3–109.2 months) none of the 26/80 patients, who did not receive inguinal irradiation, had inguinal relapse. Considered that the median time of inguinal recurrence has been reported to be around 16 months^3^, the absence of inguinal relapse in the “NIRT” group indicates that SLNB is likely to be able to identify the true negative patients.

Another relevant issue concerns the impact of advanced RT techniques in sparing the normal tissues with/without treating the inguinal LNs. In the current study, the only significantly toxicity more frequent in the “IRT” group versus “NIRT” group was the inguinal dermatitis. Interestingly, the increase of inguinal dermatitis in the “IRT” group did not translate in an increased rate of treatment suspension, neither of the duration of the suspension compared to “NIRT” group. It is important to note that the median dose to suspension was 37.7 Gy, lower in “IRT” group than “NIRT” group (34.4 Gy vs. 44.7 Gy, respectively). Genito-urinary and gastro-intestinal toxicities were similar in the two groups; in particular G3-G4 diarrhoea occurred in 3/54 and 2/26 patients, respectively.

The incidence of G ≥ 3 diarrhoea and genito-urinary toxicity in the Mitomycin-C groups reported by the two more recent phase III trials of CRT for anal cancer was 9% and 2% in the ACT II^18^, 23% and 11% in the RTOG 9811^6^, respectively. Both trials used conventional RT techniques. The use of IMRT seems to reduce the incidence of G ≥ 3 GI toxicity in a range from 0^19^ to 11–15.1%^20,21^, with most studies reporting an incidence from 7 to 9%^22,23^, without impairing the efficacy compared with traditional RT techniques^24^. A comparable range of G ≥ 3 GI toxicity, from 5 to 10%, was reported with the use of VMAT^25^ and Tomotherapy^26^. IMRT, VMAT and Tomotherapy, seem to reduce also G ≥ 3 genito-urinary toxicity to 0–3%^13,19–21,23,24,27^. Similarly to the two recent phase III trial, all the above reported trials using IMRT, VMAT or Tomotherapy, treated the inguinal LNs, then it is impossible to ascertain whether the exclusion of inguinal nodal area may translate into a further reduction of gastro-intestinal and genito-urinary toxicity.

## Conclusion

SLNB staging of inguinal LNs seems to be superior compared to the FDG-PET, it allows to better identify false positive and false negative FDG-PET patients. Combination of FDG-PET and SLNB could guide the therapeutic decision and advanced RT techniques should better avoid toxicity, especially if patients are treated on groin.
